# NORMATIVE VALUES FOR STRENGTH AND FUNCTION DERIVED FROM THE CANADIAN LONGITUDINAL STUDY ON AGING

**DOI:** 10.1093/geroni/igad104.1949

**Published:** 2023-12-21

**Authors:** Alexandra Mayhew, Hon Yiu So, Jinhui Ma, Marla Beauchamp, Lauren Griffith, Ayse Kuspinar, Justin Lang, Parminder Raina

**Affiliations:** McMaster University, Hamilton, Ontario, Canada; Oakland University, Rochester, Michigan, United States; McMaster University, Hamilton, Ontario, Canada; McMaster University, Hamilton, Ontario, Canada; McMaster University, Hamilton, Ontario, Canada; McMaster University, Hamilton, Ontario, Canada; Public Health Agency of Canada, Ottawa, Ontario, Canada; McMaster University, Hamilton, Ontario, Canada

## Abstract

Decreased muscle strength and physical function often precede disability, nursing home admission, home care use, and mortality in older adults. Normative values for commonly used physical performance-based tests are not widely available for older adults but are required for clinicians and researchers to easily identify individuals with low performance. Our objective was to develop normative values for grip strength, gait speed, Timed Up and Go, single leg balance, and five-repetition chair rise tests in a large population-based sample of Canadians aged 45 to 85 years. Baseline data (2011-2015) from the Canadian Longitudinal Study on Aging was used to estimate age- and sex-specific normative values for each of the physical tests. Participants were without disability or mobility limitation (no assistance with activities of daily living or use of mobility devices). Of the 25,470 participants eligible for the analyses 48.6% (n=12,369) were female with a mean age of 58.6 ±9.5 years. Sex-specific 5th, 10th, 20th, 50th, 80th, 90th, and 95th percentile values for each physical performance-based test were estimated. Cross-validation (n=100 repetitions) with a 30% holdout sample was used to evaluate model fit. The normative values developed in this paper can be used in clinical and research settings to identify individuals with low performance relative to their peers of the same age and sex. Interventions targeting these at-risk individuals including physical activity can prevent or delay mobility disability and the resulting cascade of increasing care requirements, health care costs, and mortality.

